# Supplementary figures and images for: Analyzing the symmetrical arrangement of structural repeats in proteins with CE-Symm
Source: PLoS Comput Biol. 2019 Apr 22;15(4):e1006842. doi: 10.1371/journal.pcbi.1006842 (PMC6504099; doi:10.1371/journal.pcbi.1006842)

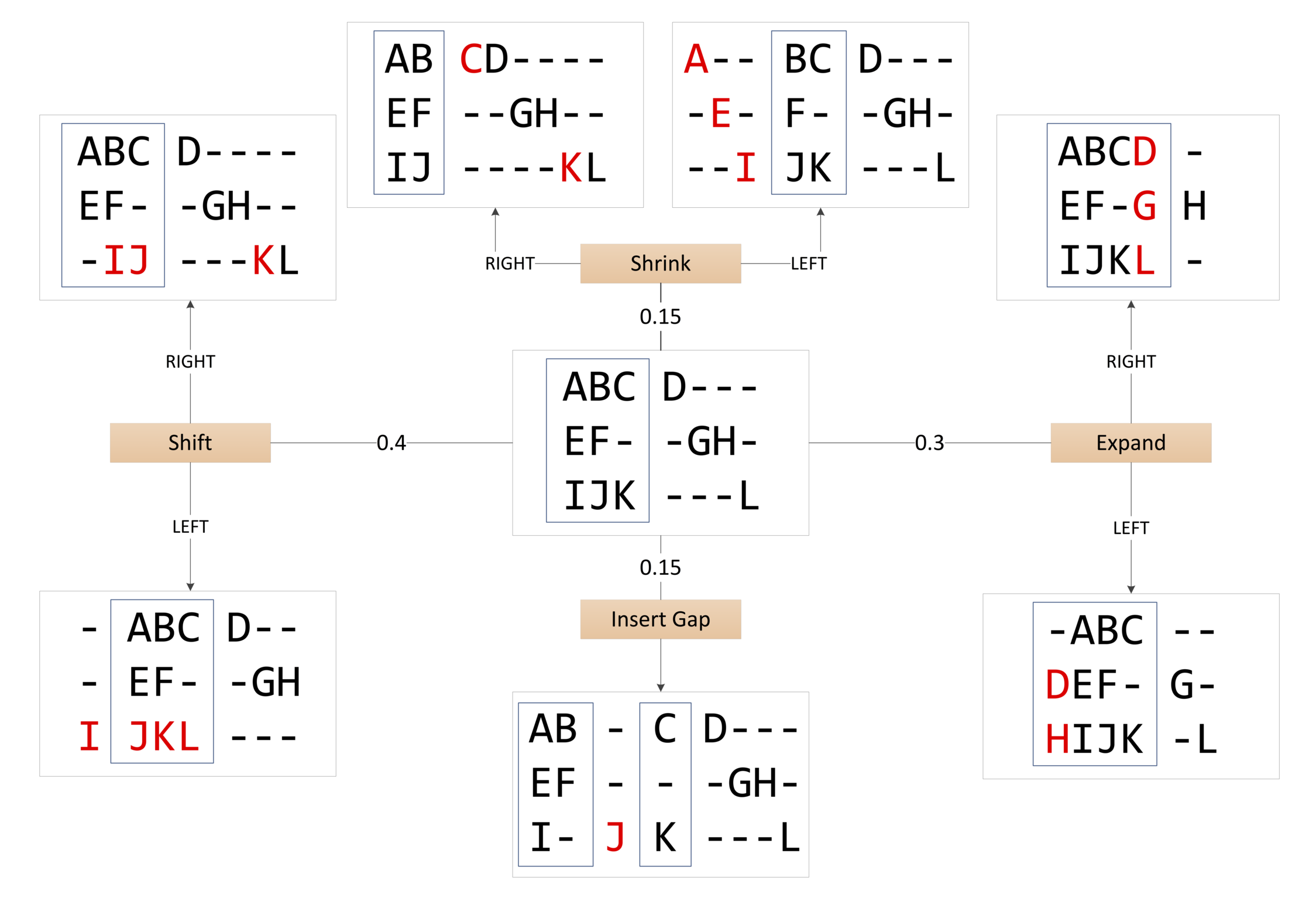

Supplement: S1 Fig — The starting alignment is shown in the center. The probability of each of the moves are indicated along the edges. (TIF) [file pcbi.1006842.s001.tif]

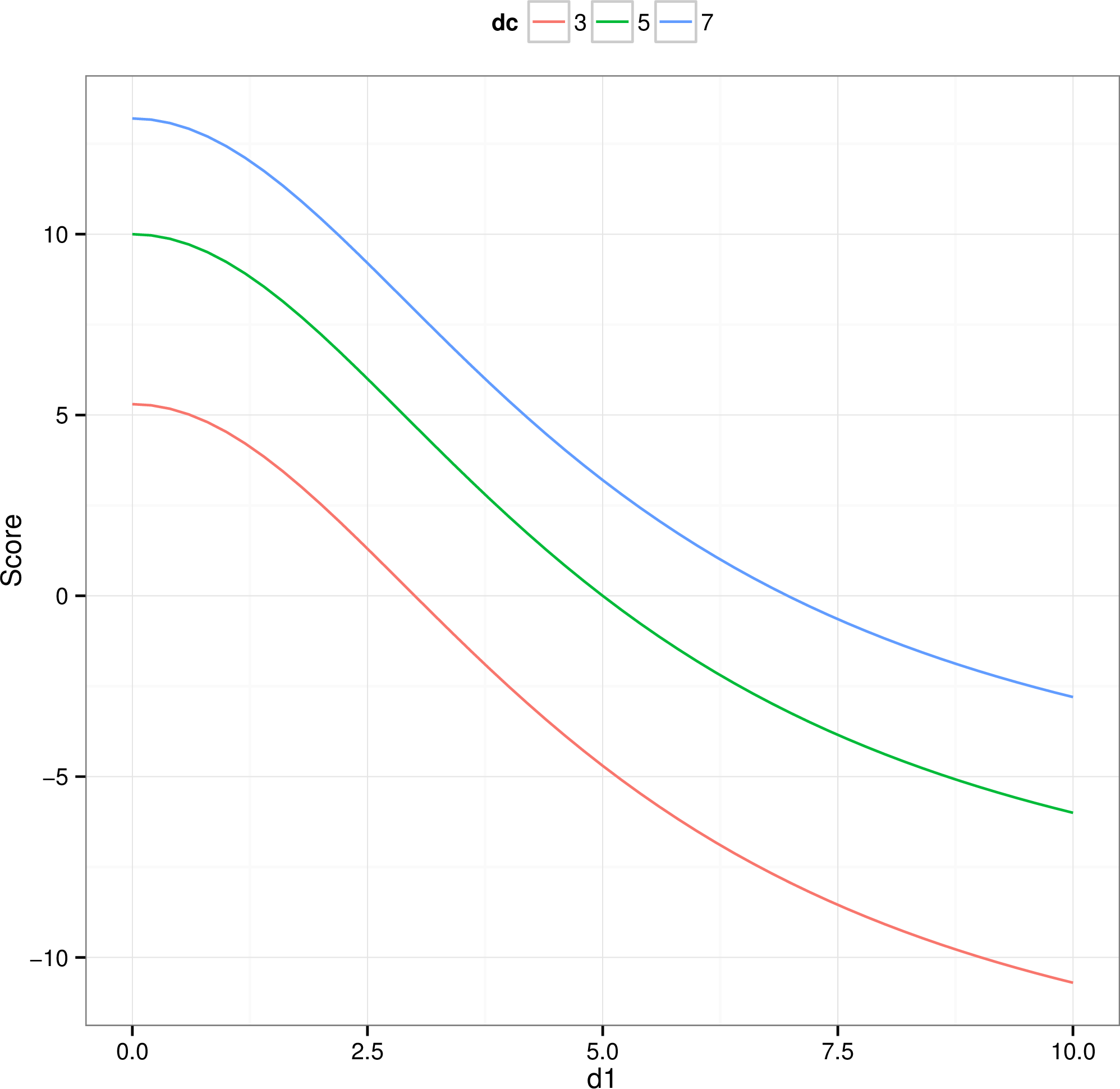

Supplement: S2 Fig — (TIF) [file pcbi.1006842.s002.tif]

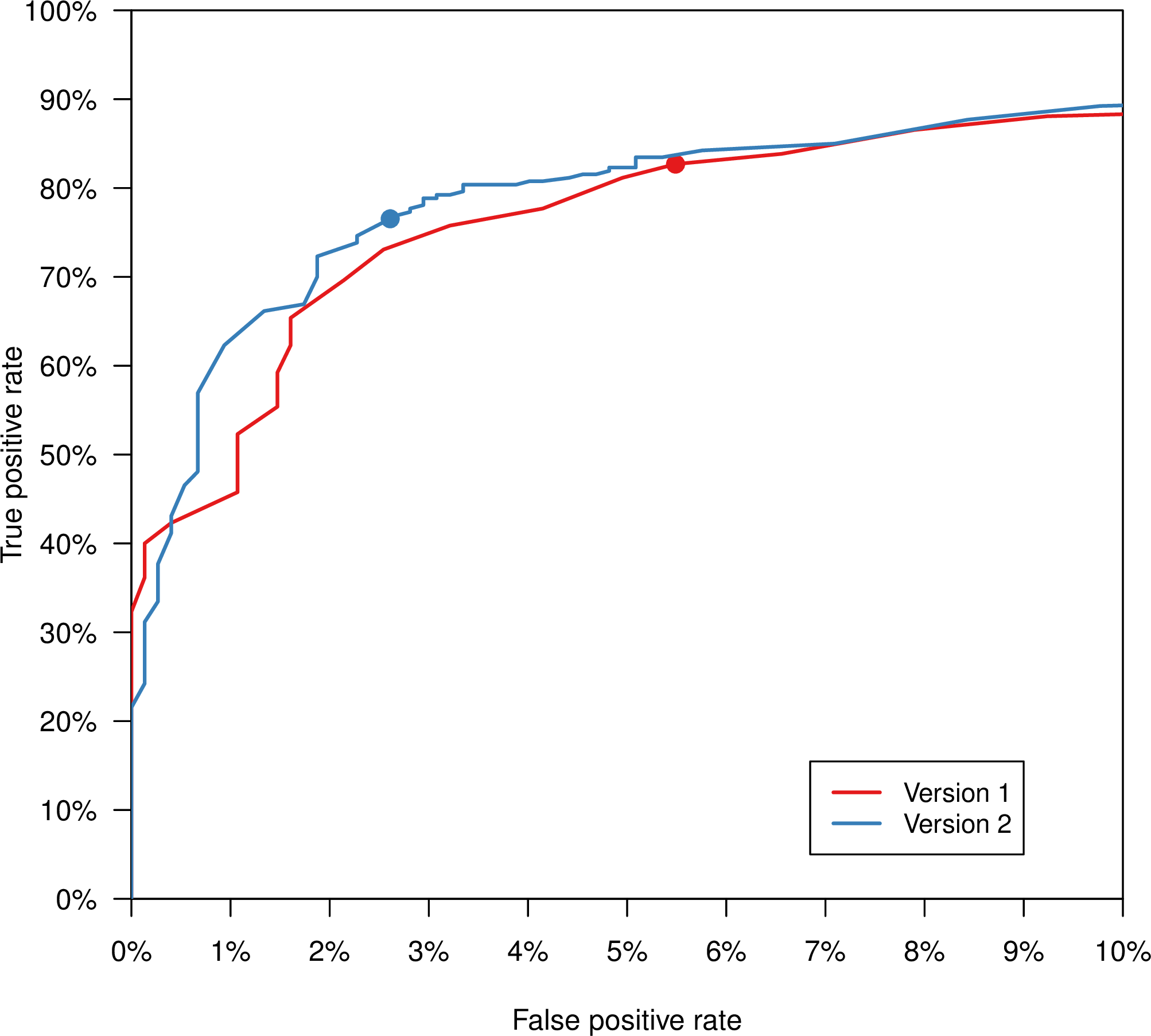

Supplement: S3 Fig — Differences in the ROC curves are not significant. The dots indicate the sensitivity and specificity at the default TM-score threshold (0.4). (TIF) [file pcbi.1006842.s003.tif]

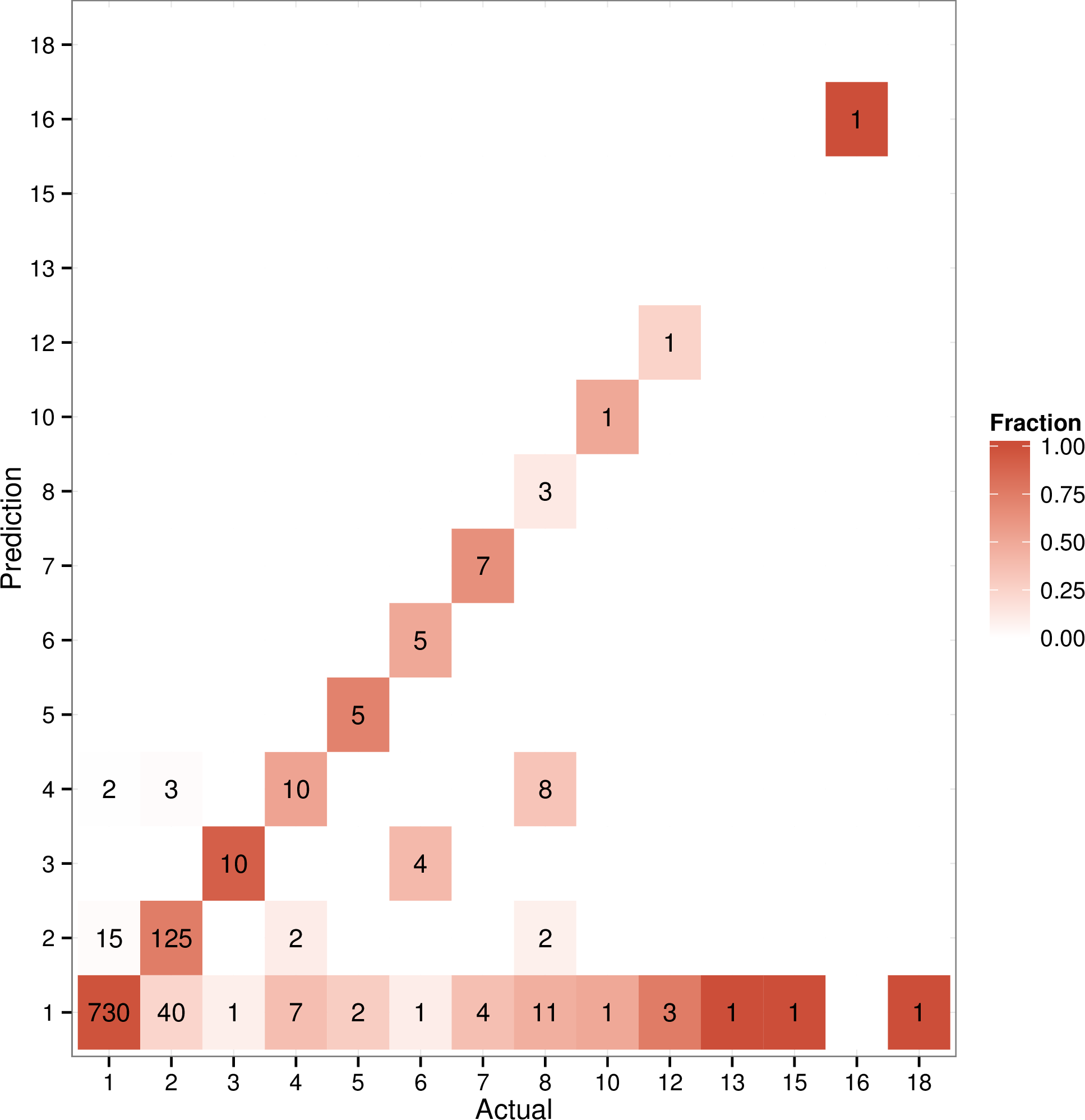

Supplement: S4 Fig — Entries of the matrix are colored by the recall of each symmetry order (columns). (TIF) [file pcbi.1006842.s004.tif]

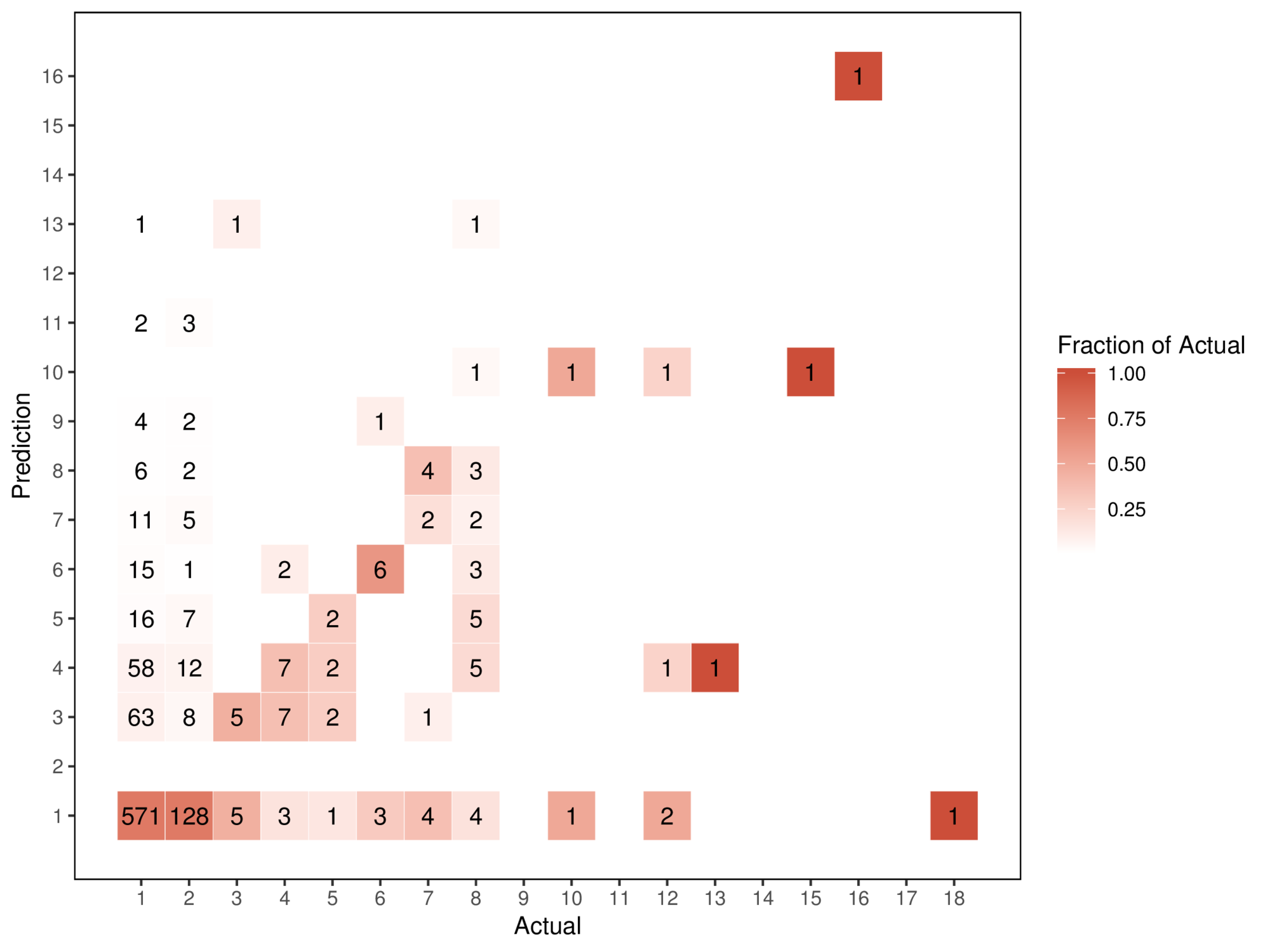

Supplement: S5 Fig — Entries of the matrix are colored by the recall of each symmetry order (columns). (TIF) [file pcbi.1006842.s005.tif]
