# Supplementary material for: Analyzing the symmetrical arrangement of structural repeats in proteins with CE-Symm
Source: PLoS Comput Biol. 2019 Apr 22;15(4):e1006842. doi: 10.1371/journal.pcbi.1006842 (PMC6504099; doi:10.1371/journal.pcbi.1006842)
Supplement: S1 Table — PDF file with the table summarizing the frequency of each type of symmetry in the benchmark. (PDF) [file pcbi.1006842.s006.pdf]

Analyzing the symmetrical arrangement of structural repeats in proteins with CE-Symm [S1 Tab]

Spencer E Bliven, Aleix Lafita, Peter W Rose, Guido Capitani, Andreas Prlić, Philip E Bourne

Type	Count	Percentage
**Asymmetric**	**747**	**74.2%**
**Rotational**	**214**	**21.2%**
C2	160	74.8%
C3	10	4.7%
C4	2	0.9%
C5	3	1.4%
C6	9	4.2%
C7	10	4.7%
C8	20	9.3%
**Dihedral**	**18**	**1.8%**
D2	14	77.8%
D3	1	5.6%
D4	2	11.1%
D5	1	5.6%
**Helical**	**11**	**1.1%**
H2	9	81.8%
H3	2	18.2%
H10	1	9.1%
**Superhelical**	**2**	**0.2%**
**Repeats**	**15**	**1.5%**

S1 Tab. Summary of the updated annotations in the internal symmetry benchmarking dataset.
