# Supplementary material for: Analyzing the symmetrical arrangement of structural repeats in proteins with CE-Symm
Source: PLoS Comput Biol. 2019 Apr 22;15(4):e1006842. doi: 10.1371/journal.pcbi.1006842 (PMC6504099; doi:10.1371/journal.pcbi.1006842)
Supplement: S4 Table — PDF file giving the precision and Cramer V for each method. Precision measures the total fraction of correct predictions and Cramer V measures the correlation between actual and predicted classes. Both measures have values in the [0, 1] interval, where 1 means perfect precision and correlation. (PDF) [file pcbi.1006842.s009.pdf]

# Analyzing the symmetrical arrangement of structural repeats in proteins with CE-Symm [S4 Tab]

Spencer E Bliven, Aleix Lafita, Peter W Rose, Guido Capitani, Andreas Prlić, Philip E Bourne

| Method | Precision | Cramer V |
|---|---|---|
| GraphComponent | 0.598 | 0.652 |
| DeltaPosition | 0.783 | 0.728 |
| RotationAngle | 0.754 | 0.642 |

**S4 Tab. Performance measures of the symmetry order detection methods for domains in the benchmark dataset with closed symmetry.** Precision measures the total fraction of correct predictions and Cramer V measures the correlation between actual and predicted classes. Both measures have values in the [0,1] interval, where 1 means perfect precision and correlation.
