# Supplementary material for: Analyzing the symmetrical arrangement of structural repeats in proteins with CE-Symm
Source: PLoS Comput Biol. 2019 Apr 22;15(4):e1006842. doi: 10.1371/journal.pcbi.1006842 (PMC6504099; doi:10.1371/journal.pcbi.1006842)
Supplement: S1 Text — PDF file with Supplementary Methods. (PDF) [file pcbi.1006842.s010.pdf]

# Analyzing the symmetrical arrangement of structural repeats in proteins with CE-Symm [S1 Text]

Spencer E Bliven, Aleix Lafita, Peter W Rose, Guido Capitani, Andreas Prlić, Philip E Bourne

## Supplemental Methods

### Order detection and refinement methods

The RotationAngle is a geometric method for determining the order in cases of closed symmetry. It is based on the angle of rotation, which can be calculated from the superposition operator (see Additional file 6 of [1]). The distance between a measured angle of rotation, $\theta$, and the closest theoretical angle of rotation for order $k$ is given by a triangle wave of frequency $k$:

$$\delta(\theta, k) = \frac{2\pi}{k} \left| \left( \left| \frac{\theta k}{2\pi} - \frac{1}{2} \right| \bmod 1 \right) - \frac{1}{2} \right| \tag{1}$$

Note that the equation given in [2] (there notated $\varepsilon(\theta)$) assumed that the ideal rotation would be $\frac{2\pi}{k}$ and neglected to account for other multiples of this. The triangle wave equation above properly accounts for the multiple possible ideal angles in rotationally symmetric structures.

The best-fit order is then the $k$ that minimizes this distance, up to some maximum order.

Both the GraphComponent and DeltaPosition methods can be understood as operations on a directed graph, where the set of residues form nodes and are connected by an edge if the alignment aligns one onto the other. Order detection is performed on the initial graph, while refinement consists of modifications of the graph. The initial self-alignment has few restrictions other than all nodes having out-degree of at most one; the presence of a circular permutation in the self-alignment permits several residues to align to a single target residue. Refinement is complete when the remaining nodes consist of either linear paths (open symmetry) or simple cycles (closed symmetry), each with $k$ nodes. These are then sorted according to the protein sequence and converted into columns of the output multiple alignment.

For the GraphComponent method, the order is first determined as the most frequent size of connected component. In the refinement step, the graph is then modified by discarding all nodes not belonging to a path of $k$ nodes. The largest subset of the remaining paths is chosen such that the sequence order of the protein is preserved in the multiple alignment. This is done by checking whether a pair of paths are "compatible", meaning they can be sorted $a < b$ such that $a_1 < b_1 < a_2 < b_2 < \cdots < a_k < b_k$. The connected component which is compatible with the most other components is selected greedily for inclusion in the refined multiple alignment. While this procedure reduces the alignment length, it was found to usually leave sufficient columns to seed the optimization step.

The DeltaPosition method attempts to better handle difficult cases with closed symmetry, where errors in the self-alignment can lead to the alignment graph becoming highly connected. For each node $x$, let $f^k(x)$ denote the node reached by following the path from $x$ through $k$ nodes. In a good alignment with the correct order $k$, many

paths will form cycles of $k$ nodes, so $f^k(x) = x$. In noisier alignments, $f^k(x)$ will not close a cycle, but will still be close to $x$ in terms of the sequence position. Thus, the position distance $\Delta(x) = \left|x - f^k(x)\right|$ measures how close a particular residue is to forming a cycle. To determine the order, $k$ is chosen up to a maximum order (8 by default) so that it minimizes

$$\sqrt{\sum_x \Delta(x)^2}. \quad (2)$$

After detecting the order, the DeltaPosition refines the alignment until it consists only of cycles of $k$ nodes. In each step, a linear path of $k$ nodes is selected from the graph to become a closed cycle. The path is chosen greedily according to the $\Delta(x)$ of it's start node, and the outgoing edge from the $k$th node is modified to point back to $x$. During every step, only cycles are considered which are compatible with previously selected cycles with respect to the protein sequence order. Finally, any nodes not belonging to cycles are discarded and the refined multiple alignment corresponding to the set of cycles is output.

Several additional order detection variants were considered during the development of `CE-Symm 2.0` but discarded after analyzing their performance [3].

### RepeatDB-lite comparison

RepeatDB-lite was run on the 1007 domains of the benchmark. Five domains produced errors since they are low resolution structures with only CA atoms positioned. In all cases, higher resolution structures of the same proteins are available which include all atoms. Thus, the following five cases were substituted into the benchmark. (`CE-Symm` results are the same on both low and high resolution structures for all cases.)

| Benchmark Domain | Replacement Structure |
|---|---|
| d1i95b_ | d2uubb1 |
| d1i96v_ | 5LMN.X:83-169 |
| d2rdo81 | d1isea_ |
| d2rdow1 | d2gycu1 |
| d3b5zd2 | d3b60d2 |

All substitutions have identical sequence and have very similar structures. SCOPe 2.01 domains were used where possible. The exception to this is `d1i96v_`, of which no other structures had been classified by SCOPe.

## References

1. Kim C, Basner J, Lee B. Detecting internally symmetric protein structures. BMC bioinformatics. 2010;11:303. doi:10.1186/1471-2105-11-303.

2. Myers-Turnbull D, Bliven SE, Rose PW, Aziz ZK, Youkharibache P, Bourne PE, et al. Systematic detection of internal symmetry in proteins using CE-symm. Journal of Molecular Biology. 2014;426(11):2255–2268. doi:10.1016/j.jmb.2014.03.010.

3. Bliven SE. Structure-Preserving Rearrangements: Algorithms for Structural Comparison and Protein Analysis. University of California San Diego; 2015. Available from: `https://escholarship.org/uc/item/0t54p4gj`.
